# Scoring Systems May be Effective in Predicting Mortality Associated with Palliative Emergency Gastrointestinal Surgery: A Retrospective Observational Study

**DOI:** 10.1007/s00268-021-06170-9

**Published:** 2021-05-31

**Authors:** M. Laitamäki, I. Alamylläri, M. Kalliomäki, J. Laukkarinen, M. Ukkonen, E. Junttila

**Affiliations:** 1 Department of Gastroenterology and Alimentary Tract Surgery, Tampere University Hospital, Tampere, Finland; 2 Faculty of Medicine and Health Technology, Tampere University, Tampere, Finland; 3 Department of Anaesthesiology and Intensive Care Medicine, Tampere University Hospital, Tampere, Finland

## Abstract

**Background:**

Palliative emergency gastrointestinal surgery is associated with significant morbidity and mortality and weighing up the benefits and harms during the decision-making may be challenging. There are very few studies on surgery in palliative patient population. The aim of this retrospective study was to evaluate morbidity and mortality after palliative emergency gastrointestinal surgery and the usability of scoring systems in predicting the outcome.

**Methods:**

Consecutive adult patients undergoing palliative emergency surgery at a tertiary hospital during the period 2015 to 2016 were included. Pre- and post-operative functional status, morbidity and mortality of patients were assessed. The predictive value of the American Society of Anesthesiologists (ASA) classification, the American College of Surgeons National Surgical Quality Improvement Program Surgical Risk Calculator (ACS NSQIP SRC) and Palliative index (PI) in estimating morbidity and mortality were determined.

**Results:**

A total of 93 patients (age 69 [28–92] years, 51% female) were included. Typical indications for surgery were bowel obstruction (52%) and securing food intake (30%). Pre-operatively two patients (2.2%) were totally dependent in daily activities, while post-operatively the respective share was 34% at discharge from hospital. The incidence of post-operative complications was 37% and 14% died during the hospital stay. One-, three-month and one-year mortality rates were 41%, 63% and 87%, respectively. While ASA score, PI score and ACS NSQIP did not predict post-operative morbidity, both ASA score and ACS NSQIP SRC predicted post-operative mortality.

**Conclusions:**

Palliative emergency laparotomy is associated with significant post-operative mortality and morbidity. Scorings, such as ASA score and ACS NSQIP SRC predict mortality in this patient population.

## Introduction

Palliative surgery aims to alleviate symptoms and improve quality of life in patients with incurable diseases. Intra-abdominally this often refers to obstruction or perforation in the gastrointestinal tract. Severe pain, nausea or constipation, inability to eat, or imminent bowel-ischemia may necessitate emergency surgery. In such situations, operations such as gastrointestinal bypass, bowel resection, intestinal anastomosis or various types of stoma are most commonly performed. [1–3]. Such palliative emergency interventions pose a challenge in terms of fast decision-making with these already fragile patients. Data on the post-operative recovery of these patients and the benefits of these interventions are scarce. Many studies focus on one type of cancer, or one procedure, while extensive studies on palliative patients in general do not exist and only few papers focus on emergency operations.

Surgical risk scores are developed for predicting the benefits and harms of the planned operation. One of the most commonly used scoring systems for predicting perioperative risks is the American Society of Anesthesiologists Physical Status Classification System (ASA) [4, 5]. Other frequently used risk scoring systems include the American College of Surgeons National Surgical Quality Improvement Program (ACS NSQIP) Surgical Risk Calculator (https://riskcalculator.facs.org/RiskCalculator) and the Palliative Index (PI) [6, 7]. ACS NSQIP is operated by means of a calculator into which 20 pre-operative patient predictors and the planned procedure are entered. The calculator predicts the risk of 18 different postoperative outcomes within 30 days following surgery while also presenting the average risk for each outcome for the given operation to compare with the patient’s risk [6]. No research has been presented on the risk scoring focusing on palliative procedures. The Palliative index (PI) is simple indicator published by Roses et al. [7]. In 2014 to predict outcomes in cancer patients undergoing emergency abdominal surgery.

The aims of the present study were to evaluate the incidence, indications and type of palliative emergency laparotomies, combined with the post-operative morbidity, motality and outcomes of these patients. The predictive value of surgical risk scores (ASA, ACS NSQIP surgical risk score and PI) were also assessed.

## Methods

**Fig. 1:**
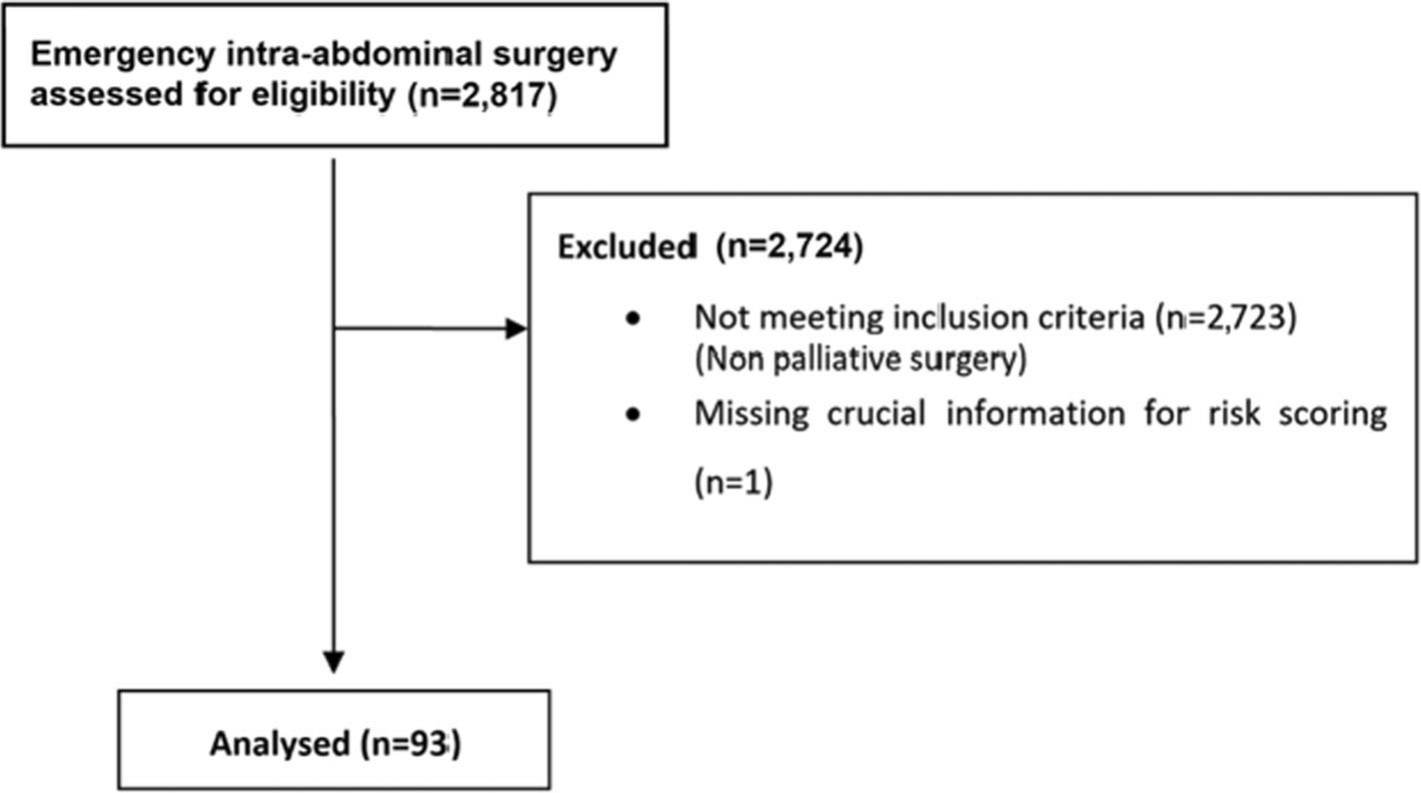
Flowchart of the study

This was a retrospective study of palliative intra-abdominal emergency operations performed in tertiary hospital, Tampere University Hospital, Finland in the period January 1, 2015 to December 31, 2016**.** The study was conducted according to the Helsinki Declaration. In compliance with the principles of the local ethics committee, exemption from consent was obtained as the data had already been collected for clinical purposes. Medical records of all emergency intra-abdominal surgeries performed during the study period were reviewed. Eligible patients were identified by searching the surgical database (URANUS; CGI Inc, Montreal, Quebec, Canada) for all those patients who had undergone laparotomy. Finally, patients with either planned palliative intra-abdominal surgery or those converted to this during the operation were included in this study as described in Fig. 1.

Patient data were collected from the Tampere University Hospital surgical database and the medical records. Recorded patient characteristics were age, sex, comorbidities, type of malignancy, ASA classification and indication for the operation. The best functional status in daily activities during the previous 30 days pre-operatively was assessed and classified into three: “independent”, “partially dependent”, and “totally dependent”, as in ACS NSQIP surgical risk score. The same three-step classification for functional status as before was used to evaluate functional recovery at discharge from hospital. Post-operative recovery variables recorded were length of stay in the postoperative care unit (PACU) and morbidity. Complications were defined and graded according to the Clavien-Dindo (C-D) classification [8]. Length of stay (LOS) in the hospital and readmissions to hospital were recorded. Hospital mortality, overall mortality and survival time were noted. Mortality was extracted from the Population Register Center. In addition, patient data was gathered for the ACS NSQIP surgical risk calculator [6].

Summary measurements were expressed as means with standard deviations or as medians with 25th–75th percentile unless otherwise stated. Continuous variables were analyzed using Student’s t-test or Mann–Whitney U-test, the latter for non-normally distributed data. Chi-square or Fisher’s exact test was used for categorical variables. Two-tailed *p* values were reported, and a *p* value < 0.05 was deemed statistically significant. The association between risk scores and morbidity was assessed comparing the probability of a serious complication with the complications classified as C-D classes 3–4, and the risk score probability of any complication with all complications included in the data. The associations between risk scores and mortality were assessed against mortality in hospital, within 14 and 30 days of discharge and in the first year thereafter. ASA class was divided into tripartite ASA 1–2, 3 and 4–5 to ensure sufficiently equal groups for comparison. ROC curves were used to visualize the performance of ASA, ACS NSQIP and the palliative index for 14, 30, 90 and 360-day mortality and morbidity. To evaluate the resolution of the test area under curve (AUC) was calculated. Statistical analyses were performed using SPSS Statistics version 22 for Windows (IBM Corp, Armonk, NY, USA).

## Results

A total of 93 patients (median age 69 [28–92] years, 51% female) were included in the study. 89 of patients had a malignant disease, three patients had acute mesenteric ischemia and one patient had complications after vascular surgery. Post-operative 90 days mortality was 63%. Age, ASA classification and coronary artery disease were associated in 90-day mortality with the statistical significance (*p* < 0.05). Pre-operatively 65% of patients were independent in daily activities, 33% were partially independent and 2.2% were totally dependent of help in daily activities. The most common indication for surgery was bowel occlusion (52%) and securing food intake (30%, including gastrojejunostomies and gastrostomas). The majority of patients (88%) had significant concomitant conditions (ASA classes III or more). Co-existing diseases were slightly more common among patients who died within 90 days (69% vs. 31%, *p* = 0.094). Pre-operatively planned operations were successfully performed on 76 (82%) patients, for 17 (18%) patients the surgery plan had to be changed during surgery and the original goal was not achieved. Sixty-five (70%) patients underwent emergency surgery which was known palliative pre-operatively and a further 28 (30%) patients were emergency surgery patients whose operation became palliative during surgery for a variety of reasons. Demographic data on the study population are presented in Table 1.

**Table 1:** Demographic, operation-related data and post-operative 90-day mortality of the study population, (*n* = 93)

Variable	All patients	Died within 90-days		*p* value
		Yes	No	
Population n (%)	93 (100)	59 (63)	34 (37)	
Age, median (min–max)	69 (28–92)	71 (47–89)	63 (28–92)	0.004
Female, n (%)	47 (51)	27 (57%)	20 (43%)	0.322
Comorbidities, n (%)	67 (72)	46 (69%)	21 (31%)	0.094
Diabetes	24 (26)	18 (%)	6 (%)	0.223
Hypertension	48 (52)	34 (71%)	14 (29%)	0.082
Heart failure	7 (7.5)	5 (71%)	2 (29%)	0.707
COPD	7 (7.5)	6 (86%)	1 (14%)	0.249
Coronary artery disease	11 (12)	10 (91%)	1 (9.1%)	0.048
Malignancy, n (%)	89 (96)	55 (62%)	34 (38%)	0.121
Colorectal	33 (25)	20 (61%)	13 (39%)	0.674
Upper gastrointestinal tract	18 (19)	8 (44%)	10 (56%)	0.062
Hepatic or pancreatic	14 (15)	12 (86%)	2 (14%)	0.075
Breast or gynaecological	15 (16)	8 (53%)	7 (47%)	0.394
Other	9 (10)	7 (78%)	2 (22%)	0.478
Pre-operative functional ability, n (%)				
Independent	60 (65%)	34 (57%)	26(43%)	0.067
Partially dependent	31 (33%)	23 (74%)	8 (26%)	0.128
Totally dependent	2 (2.2%)	2 (100%)	0 (0%)	0.531
ASA physiological status, n (%)				
1–2	11 (12)	3 (27%)	8 (73%)	0.016
3–5	82 (88)	56 (68%)	26 (32%)	0.016
Indications to surgery, n (%)				
Occlusion	48 (52)	28 (58%)	20 (42%)	0.291
Securing food intake	28 (30)	16 (57%)	12 (43%)	0.408
Perforation	6 (6.5)	6 (100%)	0 (0%)	0.082
Explorative operation	4 (4.3)	4 (100%)	0 (0%)	0.293
Other	7 (7.5)	5 (71%)	2 (29%)	1.000
Planned palliative operation n (%)	65 (70)	42 (65%)	23 (35%)	0.495

COPD, chronic obstructive pulmonary disease; ASA, American Society of Anesthesiologists classification

Data collected post-operatively are presented in Table 2. Median post-anaesthesia care unit (PACU) time was 3 h 20 min (IQR 2:32–5:17) and three patients required ICU care post-operatively. Thirty-four patients (37%) suffered from postoperative complications (C–D I–II 10%, III: 13%, IV: 0%, V: 14%). Median length of post-operative hospital stay was five (range 0–23) days. Seven (7.5%) patients required reoperation, four of them had fascial rupture, one required explorative laparotomy, one had a problem with gastrostoma and one had stoma prolapse. There were 25 hospital readmissions (27%); the most typical reason to hospital readmission was high temperature or other symptoms of infection. Post-operatively at the time of discharge from hospital 11 (12%) patients were independent in daily activities, 36 (39%) were partially independent and 32 (34%) of patients were totally dependent. The 14-day, 30-day, 90-day and 1-year mortality rates were 22%, 41%, 63% and 87%, respectively.

**Table 2:** Post-operative outcomes among patients undergoing palliative surgery

Variable	
PACU time, h:min (med, IQR, max)	3:20 (2:32–5:17, 22:35)
Post-operative ICU care, n (%)	3 (3.2)
Surgical morbidity, n (%)	34 (37)
Clavien-Dindo I-II	9 (10)
Clavien-Dindo III	12 (13)
Clavien-Dindo V (in-hospital mortality)	13 (14)
Reoperation (%)	7 (7.5)
Location for follow-up treatment, n (%)	
Home, independently	12 (13)
Home, with home nursing	6 (6.5)
Residential care home	2 (2.2)
Primary health care ward	30 (32)
Other hospital ward	30 (32)
Hospital Readmission, n (%)	24 (26)
Functional ability at discharge, n (%)	
Independent	11 (12)
Partially dependent	36 (39)
Totally dependent	32 (34)
Hospital LOS, days (med, IQR)	5 days (3–7.5)
Post op lifetime, days, (med, IQR, max)	46 (16.5–178; 971)
Mortality, n (%)	
14 days	20 (22)
30 days	38 (41)
90 days	59 (63)
1 year	81 (87)

PACU time, post-anaesthesia care unit; Post-operative ICU care, post-operative intensive care unit care, Hospital LOS length of stay

Table 3 presents the predictive values of ACS NSQIP, ASA classification and Palliative Index for post-operative morbidity and mortality. There was no statistically significant association between ACS NSQIP and any C-D class (*p* = 0.136) or serious complications (C–D 3–4) (*p* = 0.578). Nor was there any significant association between ASA and morbidity (*p* = 0.221) or C–D 3–4 morbidity (*p* = 0.547). Palliative Index was not associated with post-operative morbidity (*p* = 0.490), serious morbidity (C–D 3–4) (p = 0.904), or mortality. Both ACS NSQIP and ASA predicted mortality at 14 days, 30 days, 90 days and one year (Table 3).

**Table 3:** ACS NSQIP, ASA and Palliative index class predictive values

		No		Yes		
		Median	*Q*_1_–*Q*_3_	Median	*Q*_1_–*Q*_3_	*p* value
Morbidity *						
	ACS NSQIP	33.7	12.8–76.1	38.4	23.4–551.9	0.136
	ASA class	3	2–5	3	2–5	0.221
	Palliative index	3	2–5	3	3–5	0.490
*Clavien-Dindo 3–4*						
	ACS NSQIP	31.8	9.9–69.1	31.8	16.3–44.8	0.578
	ASA class	3	2–5	3	2–5	0.547
	Palliative index	3	2–5	3	3–5	0.904
*14-day mortality*						
	ACS NSQIP	20.1	0.7–78.8	32.8	14.3–91.2	0.004
	ASA class	3	2–5	4	3–5	0.002
	Palliative index	3	2–5	3.5	3–5	0.142
*30-day mortality*						
	ACS NSQIP	18.3	0.7–78.8	31.3	14.3–91.2	0.001
	ASA class	3	2–5	3	2–5	0.005
	Palliative index	3	2–5	3	3–5	0.812
*90-day mortality*						
	ACS NSQIP	15	0.7–65.9	31	4.9–91.2	< 0.001
	ASA class	3	2–5	3	2–4	0.003
	Palliative index	3	3–5	3	3–5	0.763
*1-year mortality*						
	ACS NSQIP	11.9	0.7–40.5	25.3	2.8–91.2	0.006
	ASA class	3	2–5	3	2–4	0.002
	Palliative index	3	2–5	3	3–5	0.648

*ASA* american society of anesthesiologists classification, *ACS NSQIP* American college of surgeons national surgical quality improvement program surgical risk calculator

^*^Clavien-Dindo I-IVb morbidity

**Fig. 2:**
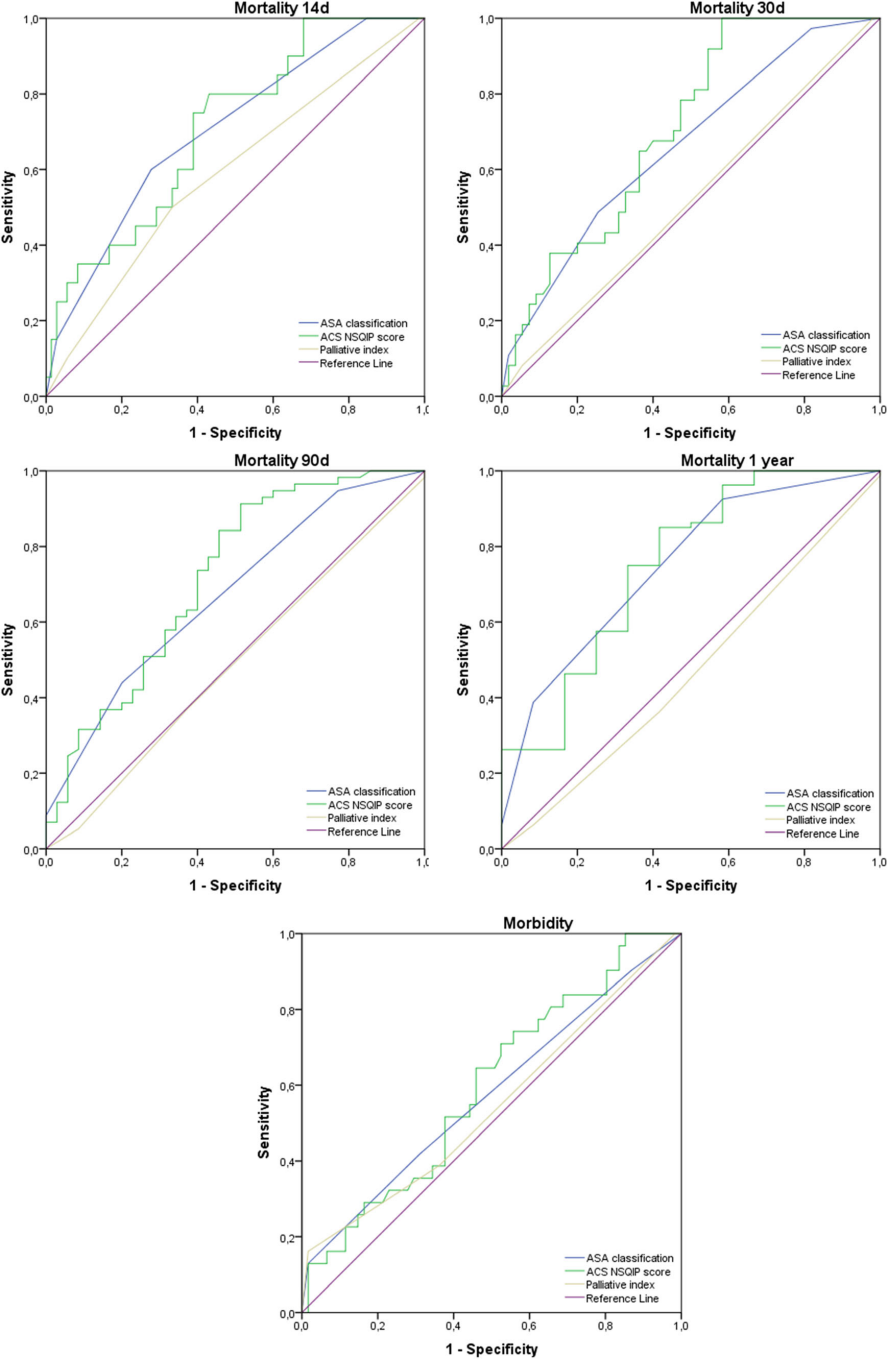
ROC curves

Figure 2 presents the ROC curves and Table 4 shows AUC values. The 14-day, 30-day, 90-day and 1-year ROC curves and area under curve (AUC) show that ACS NSQIP and ASA were reliable variables in the classification of mortality. ACS NSQIP AUC values are over 0.7, which reflects good classification. Instead, morbidity ROC curves show that ASA classification, ACS NSQIP and Palliative Index are not good at predicting complications. PI ROC curves and AUC show that PI is not good for classifying morbidity or mortality in this data.

**Table 4:** ROC AUC values

	ASA	ACS NSQIP	Palliative index
14-day mortality	.704	.710	.590
30-day mortality	.662	.707	.517
90-day mortality	.671	.718	.487
1-year mortality	.748	.749	.467
Morbidity	.575	.595	.544

*ASA* american society of anesthesiologists classification; *ACS NSQIP* american college of surgeons national surgical quality improvement program surgical risk calculator

## Discussion

The data on post-operative recovery from emergency palliative surgery is limited. According to our study results, emergency laparotomy is associated with significant short-term mortality in this study population; 22% patients died within two weeks of surgery and 63% died in 90 days. Risk scores such as ASA and ACS NSQIP do predict mortality in this study population.

According to the literature, patients undergoing palliative intra-abdominal surgery are often aged over 60 and the indication for surgery is, for example, perforation, obstruction, or internal bleeding caused by a malignity. [3, 9, 10] Our results corroborate these earlier reports. In the study, short-term mortality was considerably high and patients were extremely vulnerable, so the need for surgical treatment in this patient group needs to be considered even more carefully than normally. The conditions indicating surgery are often painful and difficult, if not unbearable, for the patient to cope with. Thus, surgical treatment may often seem a necessity. In the choice between surgery and non-operative management, it is often very difficult to evaluate the benefits, harms and other options for palliative surgery, especially for those who are not familiar with non-operative palliative care. Treatment decisions in emergency surgery are often made outside office hours and are based on subjective assessments made by on-call surgeons and anaesthetists. Clinicians should ascertain their patient’s’ advance care plan. Multidisciplinary teams including specialists in palliative care would be beneficial but are not possible outside daytime working hours their availability is limited.

In addition to multidisciplinary teams, surgical risk scores can be helpful in weighing up the benefits and harms of a procedure. In this study, both ASA score and ACS NSQIP predicted mortality among surgically treated patients, while Palliative Index did not predict mortality or morbidity. Nevertheless, these scores may provide support for surgical decision-making and informed consent. ACS NSQIP predicts the risk of 18 different post-operative outcomes within 30 days following surgery while also providing the average risk for each outcome for the given operation to compare with the risk to the patient [5]. However, while in this study both ASA scores and ACS NSQIP statistically significantly predicted mortality, they did not predict morbidity. ASA was included in this research in order to ascertain the condition of the patients prior to surgery, and to assess the predictive value of the classification in terms of post-operative mortality and morbidity. The classification alone rarely works as an indicator for operative risk as it only takes systemic diseases into account, but with other patient or operation-related factors, classification may be used as a tool in predicting post-operative risks [6].

Earlier studies have stated that there may be fewer complications related to less invasive operations, such as laparoscopic surgery than open surgery in palliative patient groups. Therefore, laparotomy should be always considered with particular care and if possible, perform less invasive operation [9, 11, 12]. However, several other factors also influence morbidity, such as ascites, diabetes, dependency in daily life, increased white cell count, type of cancer as well as how widely it has spread and obstruction located in the small intestine [13–15]. Better survival could be expected of patients with obstruction in the large intestine and who were able to eat solids at the time of discharge [13]. For instance, Burgess et al. [16] found in their research of acute laparotomies that ACS NSQIP SRC predicted most complications accurately while Parkin et al. [17] concluded that ACS NSQIP SRC was only successful in predicting mortality. Also, Collard et al. [18] showed that ACS NSQIP SRC is accurate in predicting mortality, morbidity, and serious morbidity in emergency bowel obstruction patients. [19]

Earlier studies have reported post-operative survival to vary from a few days to several years, the median usually being less than a year in palliative patient group [10, 13, 20–22]. Over one in five patients in our study population died within two weeks of surgery, and nearly 90% of patients died within one year. The pre-existing condition of a palliative patient causes an increased risk of post-operative morbidity and mortality, especially in terms of systemic complications such as pneumonia and cardiopulmonary complications.^23^ While most patients with metathesized malignancies are known to have short life expectancy, we emphasize quality of life over morbidity and mortality. In this study, only two patients were totally dependent in daily activities pre-operatively but after operation over one third were totally dependent on supportive care. Palliative patients arrive at the emergency room in poor general condition, which makes post-operative recovery challenging. Nevertheless, complications are also highly undesirable in palliative group of patients. It is possible that we surgeons are overly optimistic about the results to be achieved by performing palliative surgery. More research on post-operative quality of life and alternative non-operative care is definitely required.

Based on this study, authors recommend including NSQIP in clinical work to one of the assessments tools when evaluating palliative patients’ eligibility for operation. Another significant change in the treatment of palliative patients in Tampere University Hospital Department of Gastroenterology and Alimentary Tract Surgery was the involvement of palliative team in the treatment of palliative patient group after this study from the beginning of 2017.

The study had some limitations. First, the study was a single-center retrospective study. However, all the variables used in this study, such as those used in scoring systems (including ASA score), were registered during the hospital stay before the surgery. Second, there may have been some patient selection bias, as some of the most morbid patients may not have undergone surgery. The choice between operative and non-operative care was often based on subjective assessment often made by the surgeon and anaesthesiologist on call. After this study we have included multidisciplinary palliative teams into decision-making process, unfortunately this works only during office hours when these are available. It should be noted, however, that only in two-third of cases the surgery was pre-operative planned palliative operation and in the remaining one-third palliative approach was selected during the surgery. We were not able to get data of hospital readmissions to other hospitals than Tampere university hospital which limited the accuracy of this information; however, all the emergency cases within the hospital district are admitted to the study hospital. The most significant strengths of the study were the comprehensive post-operative details, including complete follow-up obtained from national registries. However, accurate data on causes of death was lacking from some patients.

## Conclusion

According to this study, palliative intra-abdominal emergency surgery is associated with significant short-term mortality. Risk scores, such as ACS NSQIP and ASA score, predict higher mortality and are useful in this patient population when planning surgical treatments. We recommend a multidisciplinary approach and, whenever feasible, making an advance care plan at least for those patients with high risk of mortality. In the most morbid and vulnerable high-risk patients, an alternative non-operative approach should be considered.
